# Genome-wide identification of Shaker K^+^ channel family in *Nicotiana tabacum* and functional analysis of *NtSKOR1B* in response to salt stress

**DOI:** 10.3389/fpls.2024.1378738

**Published:** 2024-04-10

**Authors:** Guang Yuan, Tongjia Nong, Oluwaseyi Setonji Hunpatin, Chuhan Shi, Xiaoqing Su, Fangzheng Xu, Yihui Wang, Zhaoting Zhang, Yang Ning, Haobao Liu, Qian Wang

**Affiliations:** ^1^ Tobacco Research Institute, Chinese Academy of Agricultural Sciences, Qingdao, China; ^2^ Graduate School of Chinese Academy of Agricultural Sciences, Beijing, China; ^3^ College of Agriculture, Qingdao Agricultural University, Qingdao, China; ^4^ China Tobacco Shandong Industrial Co., LTD Cigar Operation Center, Jinan, China; ^5^ Xuancheng City Xuanzhou District Tobacco Industry Development Center, Xuancheng, China

**Keywords:** tobacco, Shaker K+ channel, SKOR1B, salt stress, K+/Na+ ratio

## Abstract

Soil salinization poses a mounting global ecological and environmental threat. The identification of genes responsible for negative regulation of salt tolerance and their utilization in crop improvement through gene editing technologies emerges as a swift strategy for the effective utilization of saline-alkali lands. One efficient mechanism of plant salt tolerance is maintaining the proper intracellular K^+^/Na^+^ ratio. The Shaker K^+^ channels play a crucial role in potassium absorption, transport, and intracellular potassium homeostasis in plant cells. Here, the study presents the first genome-wide identification of Shaker K^+^ channels in *Nicotiana tabacum* L., along with a detailed bioinformatic analysis of the 20 identified members. Transcriptome analysis revealed a significant up-regulation of *NtSKOR1B*, an outwardly-rectifying member predominantly expressed in the root tissue of tobacco seedlings, in response to salt stress. This finding was then confirmed by GUS staining of *ProNtSKOR1B::GUS* transgenic lines and RT-qPCR analysis. Subsequently, *NtSKOR1B* knockout mutants (*ntskor1*) were then generated and subjected to salt conditions. It was found that *ntskor1* mutants exhibit enhanced salt tolerance, characterized by increased biomass, higher K^+^ content and elevated K^+^/Na^+^ ratios in both leaf and root tissues, compared to wild-type plants. These results indicate that *NtSKOR1B* knockout inhibits K^+^ efflux in root and leaf tissues of tobacco seedlings under salt stress, thereby maintaining higher K^+^/Na^+^ ratios within the cells. Thus, our study identifies *NtSKOR1B* as a negative regulator of salt tolerance in tobacco seedlings.

## 1 Introduction

Saline soils are widespread globally, with excessive exchangeable Na^+^ being the main factor that impairs crop growth and development. Salinization is typically measured by electrical conductivity (dS/m), with levels above 8 dS/m classified as moderately saline (Zaman et al., 2018). The escalating severity of soil salinization contributes to substantial losses in global agricultural production. An estimated 20% of arable land and about 33% of irrigated land globally are affected by salt stress (Meena et al., 2021). According to the Food and Agriculture Organization (FAO), under moderate salinization conditions, with soil salinity ranging from 8 to 10 dS/m, yields of maize, wheat, and cotton are reduced by 55%, 28%, and 15%, respectively (Zaman et al., 2018). Developing crop varieties adapted to saline soil conditions is a key strategy in combating soil salinization (Zörb et al., 2018).

In soils with high salinity levels, crops simultaneously experience osmotic and ionic stress (Pantha and Dassanayake, 2020; Zhao et al., 2020). Initially, higher salinity levels increase soil osmotic pressure, hindering water uptake by plant roots (Kaur and Nayyar, 2015). Additionally, excessive Na^+^ cause ionic toxicity, disrupting cellular metabolism. This disruption triggers reactive oxygen species production, leading to lipid peroxidation in cell membranes or membrane proteins. Such damage impairs the structural and functional integrity of the cell membrane, ultimately restricting plant growth and, in severe cases, resulting in plant death (Miller et al., 2010). Additionally, the excessive presence of Na^+^ and Cl^-^ inhibits the plant’s ability to absorb essential nutrients such as K^+^, Ca^2+^, Mg^2+^, and HPO_4_^2-^, leading to nutrient deficiencies in crops (Isayenkov and Maathuis, 2019).

Throughout evolution, plants have developed various mechanisms to adapt to increased salt stress. Plants can reduce Na^+^ uptake through selective root absorption or sequester excess Na^+^ in central vacuoles of cells in roots, stem bases, nodes, leaf sheaths, and older leaves (Hossain et al., 2017; Shi, 2023). Furthermore, plants maintain intracellular ionic balance by actively absorbing ions such as K^+^ from their surroundings or synthesizing organic osmolytes for osmotic regulation. (Hasegawa, 2013). Additionally, plants achieve cellular ionic homeostasis by exchanging Na^+^, K^+^ and H^+^, a process facilitated by specialized transport proteins, including High Affinity K^+^ Transporters (HKTs) and Sodium/Proton Antiporters (NHXs)(Hauser and Horie, 2010; Han et al., 2018); Keeping a stable K^+^/Na^+^ ratio in cells is a crucial strategy for plant salt tolerance (Cuin et al., 2008; Zörb et al., 2018). Studies have shown that salt-tolerant barley and alfalfa cultivars consistently maintain a higher K^+^/Na^+^ ratio in their root systems (Chen et al., 2007; Smethurst et al., 2008). Additionally, salt-tolerant barley and wheat cultivars show reduced K^+^ efflux under saline conditions, resulting in a higher K^+^/Na^+^ ratio (Wu et al., 2013, 2014). Therefore, a stable K^+^/Na^+^ ratio is a significant indicator of plant salt tolerance.

Identifying negative regulatory genes associated with salt tolerance is crucial for deciphering the mechanisms of plant responses to salt stress, and has emerged as a focal point of recent research. Transcription factors serve as central regulators and molecular switches within the intricate networks of salt stress signal transduction. The overexpression of the zinc finger transcription factor *MtZPT2-2* diminishes salt tolerance in Medicago truncatula. This factor can directly bind to the promoters of high-affinity potassium transporters *MtHKT1;1* and *MtHKT1;2*, inhibiting their expression, leading to Na^+^ imbalance and reduced activity of antioxidant enzymes (Huang et al., 2024). *CONSTANS* (CO), a pivotal transcription factor in the flowering pathway, adversely affects salt tolerance in Arabidopsis. Mutants with a loss of CO function exhibit enhanced salt tolerance compared to wild-type plants (Du et al., 2023). Protein phosphorylation, mediated by kinases, is crucial in the signal transduction pathways responding to salt stress. In rice, the transcription factor *OsWRKY53* acts as a negative regulator, controlling the expression of the *OsMKK10.2* kinase, impacting root Na^+^ balance, and inhibiting the sodium transporter *OsHKT1;5*, thereby orchestrating the defense mechanisms against ion stress (Yu et al., 2023). Silencing the calmodulin gene *HvCAM1* in barley through RNAi enhances the plant’s salt tolerance. Overexpressing *TaCIPK25* (Cbl Interacting Protein Kinases) in wheat increases Na^+^ sensitivity, disrupts root cell Na^+^/H^+^ exchange, and results in excessive Na^+^ accumulation. Additionally, certain proteins directly exert negative regulatory functions in relation to salt stress. Transgenic apple calli overexpressing *MdGRF6* displayed heightened sensitivity to salt stress, whereas *MdGRF6*-RNAi transgenic calli demonstrated increased salt tolerance (Jin et al., 2016). These investigations uncover the intricate mechanisms of plant response to salt stress, highlighting the critical role of negative regulators in modulating salt tolerance.

Shaker K^+^ channels play a crucial role in K^+^ absorption, transport, and the regulation of intracellular K^+^ balance in plants (Véry et al., 2014). Numerous studies have demonstrated that Shaker potassium channels regulate plant salt tolerance through the modulation of the K^+^/Na^+^ ratio. Recent research indicates that the soybean Shaker inward rectifier potassium channel gene *GmAKT1* is significantly upregulated under salt stress conditions. Overexpressing *GmAKT1* in yeast and Arabidopsis akt1 mutants compensates for their low K^+^ condition deficits. Under salt stress, transgenic Arabidopsis and soybean plants overexpressing *GmAKT1* exhibit improved growth, increased K^+^ concentration, decreased Na^+^ concentration, and a reduced Na^+^/K^+^ ratio. Further analysis revealed that *GmAKT1* overexpression significantly boosts the expression of genes involved in plasma membrane absorption and transport such as *GmsSOS1*, *GmHKT1*, and *GmNHX1*, enhancing K^+^ uptake, facilitating Na^+^ exclusion, maintaining intracellular ion equilibrium, and augmenting salt tolerance (Wang et al., 2021). The rice Shaker outward rectifier potassium channel homolog gene *OsK5.2* regulates stomatal closure in leaf guard cells, reducing water transpiration and Na^+^ transport to leaves, and enhances K^+^ secretion into the xylem sap in roots, thus maintaining and enhancing K^+^ transport under salt stress conditions. The *OsK5.2* knockout mutant exhibited increased sensitivity to salt stress, further affirming *OsK5.2*’s critical role in regulating rice salt tolerance (Zhou et al., 2022). Previous research has indicated that Shaker potassium channels are involved in salt stress response through their impact on K^+^/Na^+^ balance. However, specific members that act as negative regulators of salt tolerance have yet to be identified.

Tobacco, a classic model crop, has had its genome fully sequenced (Sierro et al., 2014). However, studies focusing on the functionality of the Shaker K^+^ channel family in tobacco, particularly in relation to resistance to salt stress, are limited. This study delineates the genome-wide identification of the Shaker K^+^ channels in tobacco, examining its members, conserved domains, and promoter elements to establish a groundwork for subsequent investigations into the gene family’s potential biological functions within tobacco. Transcriptome data revealed a gene upregulated by salt stress, and gene editing technology confirmed its role in negatively regulating tobacco’s salt tolerance. This enhances our comprehension of plants’ response mechanisms to salt stress through the regulation of K^+^/Na^+^. By elucidating this protein’s role in K^+^ and Na^+^ regulation, this research not only identifies a potential target for enhancing crop salt tolerance but also offers a viable approach for the holistic use of saline soils.

## 2 Materials and methods

### 2.1 Identification of Shaker K^+^ channel family genes in tobacco

Protein sequences for the nine *Arabidopsis thaliana* Shaker family members were obtained from the TAIR database (https://www.arabidopsis.org/).These sequences were then used as queries for a subsequent BLAST search in the NCBI database (https://www.ncbi.nlm.nih.gov/) to identify Shaker K^+^ channel family in tobacco (*N. tabacum*), using an E-value threshold of 1e^-5^. Redundant sequences were manually removed. The redundant entries caused by alternative splicing were removed manually, and the identified sequences were then used as new queries to do a second BLASTP search. The sequences without Shaker K^+^ channel family-specific motifs were removed after sequence alignment and SMART analysis (http://smart.embl-heidelberg.de/). The molecular weight, isoelectric point, and amino acid number of proteins were calculated with the ExPASy tool (https://web.expasy.org/protparam/).

### 2.2 Analysis of protein sequences and promoter elements of tobacco Shaker K^+^ channel family

A phylogenetic tree of Shaker K^+^ channel family members from *N. tabacum*, *Arabidopsis thaliana* and *Oryza sativa* was constructed using the Neighbor-Joining method in MEGA6, under the default parameters (bootstrap replicates=1000) (Tamura et al., 2013). Multi-sequence alignment was performed using Clustal Omega (https://www.ebi.ac.uk/Tools/msa/clustalo/) and the result was presented by GeneDoc (Nicholas, 1997). InterPro (https://www.ebi.ac.uk/) was utilized for conserved domain analysis, with findings visualized in Microsoft PowerPoint. To find cis-acting elements localized in the promoter region of Shaker K^+^ channel family genes, 3000 bp of promoter regions upstream of the 5’-UTR of Shaker K^+^ channel family genes were queried. The cis-acting elements were predicted by PlantCARE (http://bioinformatics.psb.ugent.be/webtools/plantcare/html/) (Rombauts et al., 1999) and presented with a heatmap by Tbtools (Chen et al., 2020).

### 2.3 Tobacco plant cultivation, salt stress treatment, and sampling

This study used the tobacco variety Zhongyan 100 (*N. tabacum*), grown in a greenhouse under controlled conditions: 16 hours of light and 8 hours of darkness, at temperatures of 23-25°C and 70% relative humidity. Initially, tobacco seeds were sown in soil. After 20 days of germination, seedlings were transplanted onto floating rafts for hydroponic cultivation in a 1/2 Hoagland nutrient solution. Following a 6-day acclimatization period, salt treatment was administered (1/2 Hoagland solution with 100 mM NaCl), while controls received the 1/2 Hoagland solution alone. Nutrient solutions were refreshed every three days.

For the *NtSKOR1B* RT-qPCR analysis, root, stem, and leaf samples were collected at specific intervals: immediately (0 h), 6 h, 12 h, 30 h, 60 h, and 6 days following a 6-day hydroponic culture period with salt treatment (100 mM NaCl). The samples were immediately flash-frozen in liquid nitrogen and then stored at -80°C.

GUS staining experiments began with the sowing of *NtSKOR1B* promoter plants (*ProNtSKOR1B::GUS*). Following 20 days of germination and a 6-day hydroponic culture period, samples of roots, stems, and leaves were collected at designated intervals: immediately (0 h), 12 h, and 30 h after salt treatment exposure. GUS staining was then conducted using β-Galactosidase Reporter Gene Staining Ki (Beijing Leagene Biotech. Co., Ltd., Cat No./ID: DP0013, Beijing, China), with observations made post-staining.

Sampling to measure physiological indicators: Following a 6-day adaptation to hydroculture, tobacco seedlings are subjected to salt stress (1/2 Hoagland nutrient solution with 100 mM NaCl), while the control group receives only 1/2 Hoagland nutrient solution. Young tobacco plants were harvested on the 11th day post-salt stress treatment for the measurement of the following indicators. Plants from various strains and treatments were selected, focusing on the fourth leaf position (the first mature leaf post-salt treatment) to assess leaf length and width, followed by curing the entire plant at 105°C for 30 minutes and drying at 80°C until a constant weight was achieved. Biomass was weighed, n=6. Measurement of ion content: Leaves and roots were separated. Root samples were initially rinsed with ddH2O and subsequently dried with absorbent paper to eliminate external ions. Fresh samples were dried at 105°C until reaching a stable weight, followed by grinding the dried tissue into powder. Approximately 0.01 g of dry sample was placed into a test tube, to which 8 mL of 0.5 M HCl was added, and the mixture was extracted at 20°C and 150 rpm for 30 minutes. Subsequently, the mixture was filtered and adjusted to the extraction volume for testing. A standard K^+^ solution was prepared using 0.5 M HCl, and a flame spectrophotometer (6400A) was employed to quantify the ion content, n=3. Formula for calculating potassium content:


$${\text{K}}^{+}\left(\text{μg}/\text{mg DW}\right)=(\left(\text{A}/\text{M}\right)\text{Dilution factor }*0.001)/\text{m}$$

A: Concentration calculated from readings based on standard curve; M: Relative molecular mass of K^+^; V: Reading volume; m: Dry weight of sample.

### 2.4 RT-qPCR

Total RNA was isolated from tissues of young tobacco plants and purified with the RNeasy Plus Mini Kit (Qiagen, Cat No./ID: 74134) following the manufacturer’s protocol. The RT-qPCR program was described in (Mao et al., 2021). RNA was reverse transcribed into cDNA using Evo M-MLV Mix Kit with gDNA Clean for qPCR (Accurate Biotechnology (Hunan) Co., Ltd, Cat No./ID: AG11728, Changsha, China) and the cDNA was amplified using SYBR Green Pro Taq HS qPCR Premix (Accurate Biotechnology (Hunan) Co., Ltd, Cat No./ID: AG11701, Changsha, China) on the LightCycler^®^ 96 Instrument (F. Hoffmann-La Roche Ltd, Switzerland). All the primers used for RT-qPCR are listed in **Supplementary Table 1**. The amplification reactions were performed in a total volume of 10 μl, containing 5 μl 2×SYBR Green Pro Taq HS qPCR Premix, 0.6 μl forward and reverse primers (10 μM), 1 μl cDNA (10 times diluted), and 3.4 μl ddH2O. The RT-qPCR amplification program was as follows: 95oC for 10 min; 95oC for 10 s, 60oC for 30 s and amplification for 40 cycles. Analysis of the relative gene expression data was conducted using the 2^-ΔC’t^ method (Mao et al., 2021).

### 2.5 Construction and identification of *NtSKOR1B* knockout mutants

Specific primers, *NtSKOR1B*-1F and *NtSKOR1B*-1R, were designed from the LOC104103384 gene sequence for PCR amplification, using *N. tabacum* L. cDNA to obtain the *NtSKOR1B* sequence. The knockout site within the *NtSKOR1B* sequence was identified, leading to the design of knockout primers *NtSKOR1B*-Crispr-58F and *NtSKOR1B*-Crispr-58R for constructing the pORE-Cas9/gRNA-*NTSKOR1B* gene knockout vector. The pORE-Cas9/gRNA-*NtSKOR1B* construct was introduced into Zhongyan 100 using the Agrobacterium-mediated leaf disk method, generating T0 transgenic mutants. Plants were initially screened for various editing outcomes using primers *NTSKOR1B*-Crispr-9734F and *NtSKOR1B*-Crispr-10293R. Nine C1 generation lines were cultivated, and DNA from homozygous mutants was extracted for PCR amplification and sequencing with specific primers *NtSKOR1B*-Crispr-9734F and *NtSKOR1B*-Crispr-10293R. This led to the identification of two homozygous knockout lines, characterized by distinct edits: C2 with a T insertion at position 911, and C6 with an 11-base deletion starting at position 989, causing translational termination (**Supplementary Figure 1**).

### 2.6 Construction and identification of *ProNtSKOR1B::GUS* transgenic lines

Genomic DNA from plants was extracted using the CTAB method, and the *NtSKOR1B* promoter sequence upstream of the ATG start codon for LOC104103384 was obtained through PCR amplification with primers *NtSKOR1B*pro-1F and *NtSKOR1Bpro*-1R. This yielded a 2439 bp *NtSKOR1B* promoter sequence, which was then cloned into the plasmid pMD19-T-*NtSKOR1B*pro and confirmed by sequencing. Using the plasmid pMD19-T-*NtSKOR1B*pro as a template and primers *NtSKOR1B*pro-2F and *NtSKOR1B*pro-2R for amplification, the binary vector pBI101-*NtSKOR1B*pro was obtained, which drives the GUS gene under the *NtSKOR1B* promoter’s control. The Agrobacterium-mediated leaf disk method was employed for transforming this vector into tobacco plants, generating T0 transgenic lines. The T0 generation lines was confirmed positive (**Supplementary Figure 2**), using the identification primers *NtSKOR1B*pro-3F and *NtSKOR1B*pro-1R. Harvest T0 generation seeds from positive plants and sow them on 1/2 MS plates with 50 mg/L kanamycin sulfate. Following resistance screening, strictly self-cross lines displaying a 3:1 resistance ratio, selecting for resistant lines. Staining was performed on T1 generation homozygous plants.

### 2.7 Statistical analysis

Statistical analysis was done using IBM SPSS Statistics 23 software. Significant differences were examined by one-way ANOVA using the LSD test at p<0.05 and p<0.01. The figures were drawn by GraphPad Prism 6.0.

## 3 Result analysis

### 3.1 Genome-wide identification and phylogenetic analysis of Shaker K^+^ channel family in *N. tabacum*

**Figure 1:**
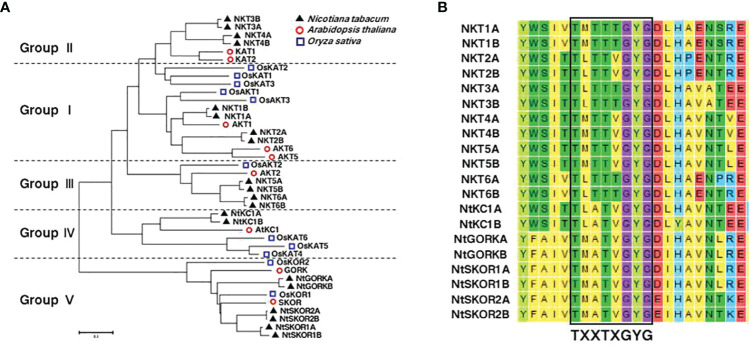
Evolutionary tree analysis and signature sequence alignment of Shaker family members in *Arabidopsis thaliana*, *Oryza sativa*, and *N. tabacum.* **(A)** Phylogenetic Analysis of *N. tabacum*, *Arabidopsis thaliana* and *Oryza sativa* Shaker K^+^ channels: Amino acid sequences for *Arabidopsis thalian*a and *Oryza sativa* were sourced from TAIR (https://www.arabidopsis.org/), NCBI (https://www.ncbi.nlm.nih.gov/), and Sol Genomics Network (https://solgenomics.net/) respectively. The phylogenetic tree was generated using MEGA6 through the Neighbor-Joining method. **(B)** The black box highlights the characteristic signature sequence TXXTXGYG, a hallmark of selective K^+^ channels.

Genomic screening identified 20 members of the *N. tabacum* L. Shaker K^+^ channel family using NCBI and SMART tools. Members were systematically numbered according to their similarity to *Arabidopsis thaliana* Shaker K^+^ channel family counterparts (**Figure 1**, **Table 1**). Ten Shaker K^+^ family members were attributed to the maternal ancestor, *Nicotiana sylvestris*, and labeled with the suffix “A”, while the remaining ten were derived from the paternal ancestor, *Nicotiana tomentosiformis*, and marked with “B”. The K^+^ channel proteins varied in length from 598 to 892 amino acids, with molecular weights ranging from 69.5 to 100.5 kDa.

**Table 1:** Fundamental characteristics of Shaker K^+^ channel family in *N. tabacum*.

New Name	Diploid Progenitors	Classification	The Name/ID of Arabidopsis based on NCBI	Representative Protein Accession or Previous Name	Gene ID in NCBI	AA	*pl*	MW(Da)
NKT1A	*N. sylvestris*	Group I	AKT1/817206	XP_009772048.1	LOC104222515	893	6.54	100508.19
NKT1B	*N. tomentosiformis*	Group I		XP_009619489.1	LOC104111487	892	7.06	100290.93
NKT2A	*N. sylvestris*	Group I	AKT6/817099	XP_009799565.1	LOC104245633	881	6.56	99362.20
NKT2B	*N. tomentosiformis*	Group I		XP_009621376.1 (NKT1)	LOC104113012	879	6.64	98765.52
NKT3A	*N. sylvestris*	Group II	KAT1/834666	XP_009804747.1	LOC104249919	682	6.77	78146.90
NKT3B	*N. tomentosiformis*	Group II		XP_009602722.1	LOC104097809	681	7.19	77910.82
NKT4A	*N. sylvestris*	Group II	KAT2/827555	XP_009760491.1	LOC104212831	713	5.96	82266.82
NKT4B	*N. tomentosiformis*	Group II		XP_009616640.1	LOC104212831	709	5.96	81884.84
NKT5A	*N. sylvestris*	Group III	AKT2/828311	XP_009795716.1	LOC104242371	864	6.71	99138.50
NKT5B	*N. tomentosiformis*	Group III		XP_009616703.1	LOC104109179	847	6.71	97218.22
NKT6A	*N. sylvestris*	Group III		XP_009791067.1	LOC104238419	837	7.33	95932.75
NKT6B	*N. tomentosiformis*	Group III		XP_009614130.1 (NKT2)	LOC104107110	824	6.71	94602.29
NtKC1A	*N. sylvestris*	Group IV	AtKC1/829400	XP_009801140.1	LOC104246930	641	7.29	73061.33
NtKC1B	*N. tomentosiformis*	Group IV		XP_018628621.1 (NtKC1)	LOC104103008	642	6.99	73381.65
NtGORKA	*N. sylvestris*	Group V	GORK/833728	XP_009771768.1 (TORK1)	LOC104222255	834	6.56	95114.16
NtGORKB	*N. tomentosiformis*	Group V		XP_009597240.1	LOC104093217	598	7.53	69487.16
NtSKOR1A	*N. sylvestris*	Group V	SKOR/821052	XP_009762658.1	LOC104214657	827	6.52	94792.21
NtSKOR1B	*N. tomentosiformis*	Group V		XP_009609577.1	LOC104103384	827	6.64	94970.55
NtSKOR2A	*N. sylvestris*	Group V		XP_009802068.1	LOC104247691	821	6.45	94205.60
NtSKOR2B	*N. tomentosiformis*	Group V		XP_009626879.1	LOC104117520	810	6.55	92899.10

To construct a phylogenetic tree (**Figure 1A**), members were selected from the Shaker K^+^ channel families in *Arabidopsis thaliana*, *Oryza sativa*, and *N. tabacum*. The results showed that, like the other two species, *N. tabacum* Shaker K^+^ channel family were categorized into five groups.

In 2007, at a time when tobacco genome data were not yet available, Toshio Sano’s team utilized Degenerate PCR and RACE technology to clone four members of the Shaker K^+^ channels from the BY-2 cell line. These channels were designated as *NKT1*, *NKT2*, *NtKC1*, and *NTORK1*, respectively (Sano et al., 2007). To avoid potential confusion and align with Arabidopsis nomenclature conventions, this study renamed these four genes *NKT2B*, *NKT6B*, *NKC1B*, and *NtGORKA*. All members of the tobacco Shaker K^+^ channel families possess the signature sequence TXXTXGYG, characteristic of selective K^+^ channels. (**Figure 1B**).

### 3.2 Analysis of the conserved domains in the members of *N. tabacum* Shaker K^+^ channel family

**Figure 2:**
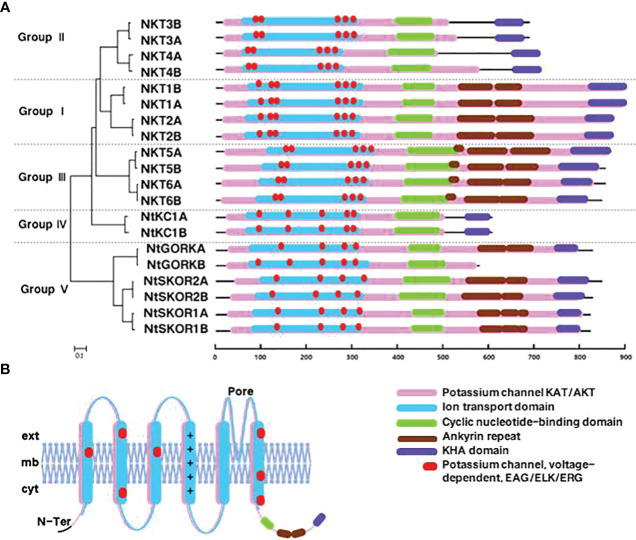
Analysis of the conserved domains in the members of *N. tabacum* Shaker K^+^ channel family. **(A)** Conserved domains analysis. **(B)** Using *NKT1B* as an example, this panel presents a schematic of the simulated transmembrane structure of a Shaker family protein. Here, “ext” represents the extracellular region, “mb” the cell membrane, “cyt” the intracellular region, and “N-ter” the N-terminal beginning of the protein.

InterPro analysis predicted six conserved domains in *N. tabacum*. Shaker K^+^ channel family members: K^+^ channel KAT/AKT domain, Ion transport domain, Cyclic nucleotide-binding domain, KHA domain, Ankyrin repeat domain, and voltage-dependent K^+^ channel EAG/ELK/ERG domain (**Figure 2**). The K^+^ channel KAT/AKT domain, present in all *N. tabacum* L. Shaker proteins, is the most fundamental conserved domain. The Ion transport domain plays a crucial role in regulating K^+^ transmembrane transport. The Cyclic nucleotide-binding and KHA domains are essential for channel function regulation, whereas the Ankyrin repeat domain primarily mediates protein-protein interactions. The voltage-dependent K^+^ channel EAG/ELK/ERG domain senses voltage changes across the cell membrane.

In Groups I, III, and V, 13 out of 14 members possess all six conserved domains mentioned, whereas Groups II and IV lack the Ankyrin repeat domain. The count of voltage-dependent K^+^ channel EAG/ELK/ERG domains varies among subgroups, with six in Group I, five in Groups II, III, and V, and six in Group IV. Variations in types, lengths, and numbers of conserved domains likely affect the proteins’ response functions.

### 3.3 Analysis of promoter elements in *N. tabacum* Shaker K^+^ channel family members

**Figure 3:**
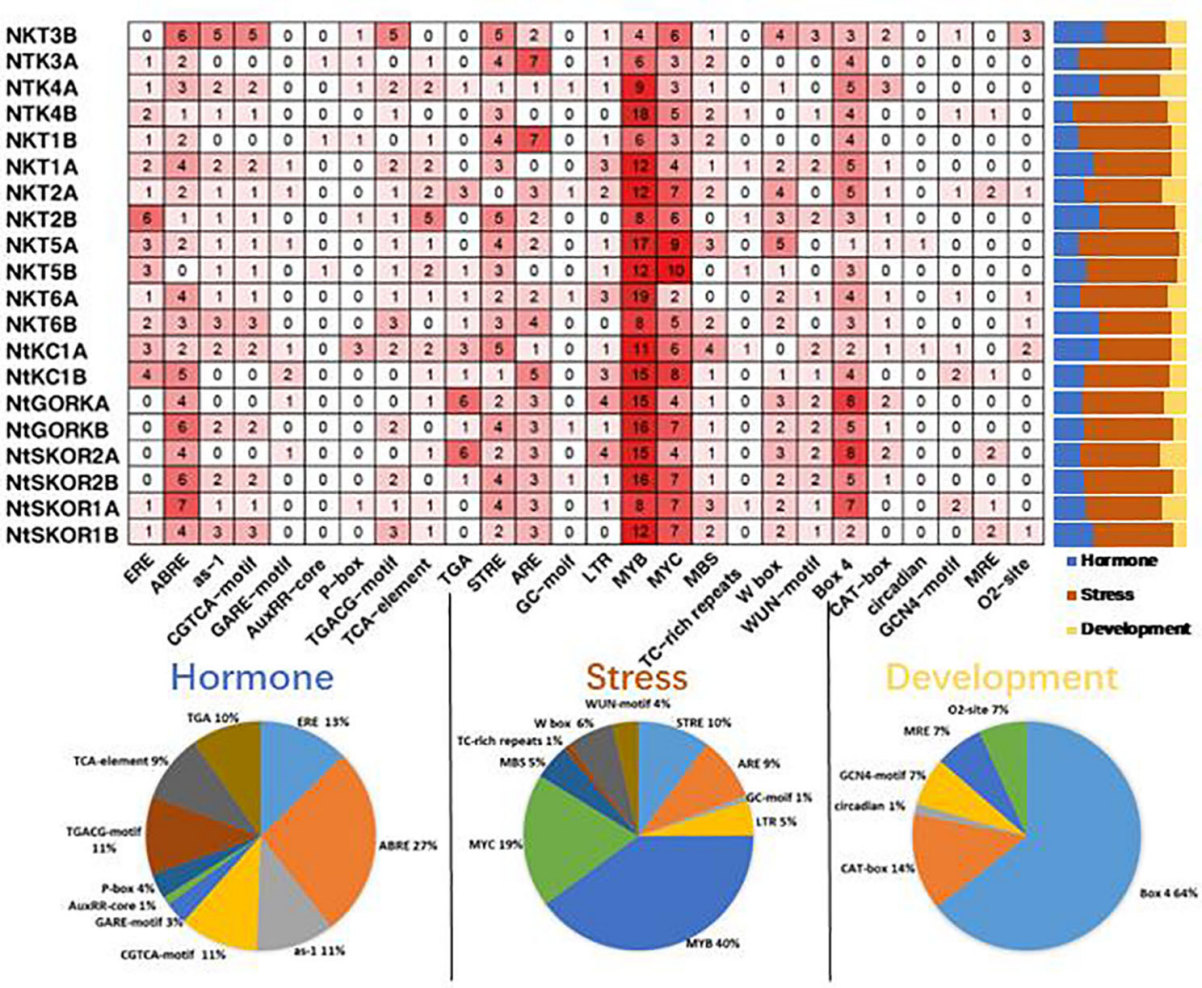
Analysis of promoter elements in *N. tabacum* Shaker K^+^ channel family members. The bar graph shows the proportion of elements related to hormone response (blue), stress response (orange), and growth and development (yellow) in the total number of elements in the promoter region of each member; the pie chart shows the proportion of a specific element in the total number of elements related to hormone response (blue), stress response (orange), and growth and development (yellow).

To elucidate the potential functions and regulatory mechanisms of Shaker K^+^ channels in *N. tabacum*, we analyzed the cis-acting elements in promoter sequences (3000 bp upstream of the ATG start codon) of 20 Shaker K^+^ channel family using PlantCARE software. Analysis revealed that Shaker K^+^ channel promoters predominantly contain cis-acting elements associated with three major biological processes: abiotic stress response, plant hormone response, and growth and development. Specifically, there are 10 types of elements each for abiotic stress and plant hormones, and 6 for growth and development. (**Figure 3**) This indicates that the Shaker K^+^ channel family significantly influences tobacco’s response to abiotic stress, hormone regulation, and growth and development processes.

Abiotic stress response elements are the most abundant in the promoter regions of *N. tabacum* Shaker K^+^ channel genes, comprising 31.58%-71.93% of all identified elements. MYB and MYC elements represent 40% and 19% of stress response elements, respectively. The antioxidant response element (ARE) was found in 19 Shaker members, making up 9% of stress elements. The drought response element (MBS) was present in 17 members (5%), and the W-box, linked to K^+^ uptake and signal transduction, was found in 16 members (6%). Phytohormone response elements constituted 10.53%-38.60% of elements. The ABA response element (ABRE) was identified in 19 Shaker members, comprising 27% of stress elements. Elements related to growth and development accounted for 5.26%-21.05% of the total, with all members containing the light-responsive element (Box 4), comprising 64% of these elements.

### 3.4 Transcriptomic analysis of *N. tabacum* Shaker K^+^ channel family members under salt treatment in roots

**Figure 4:**
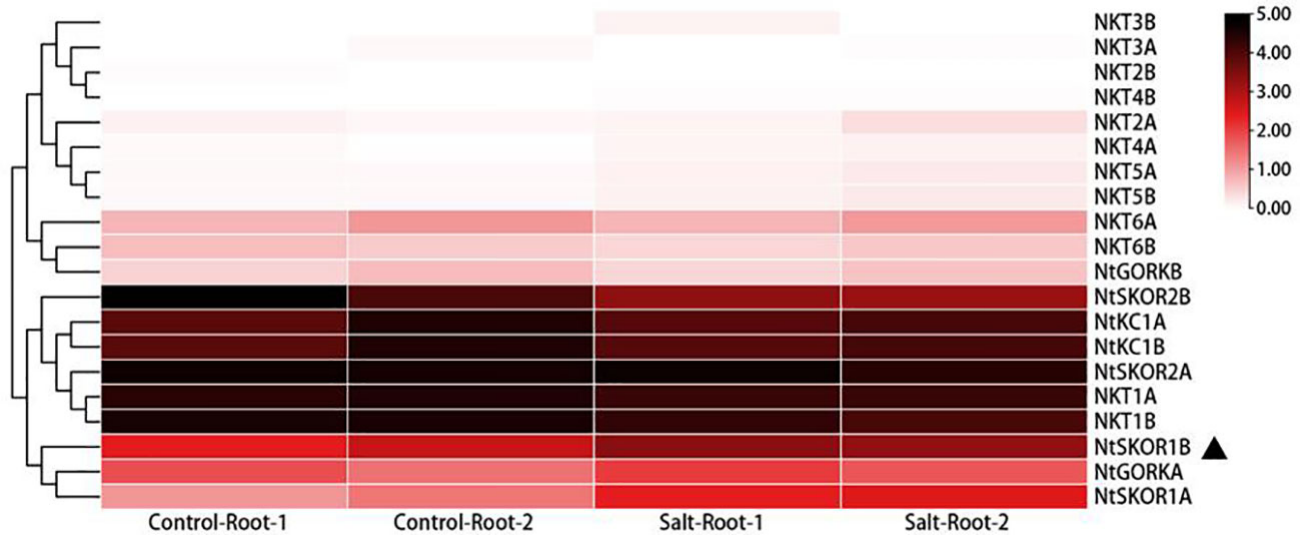
Differential expression of *N. tabacum* Shaker K^+^ channel family members in roots subjected to salt stress. “1” signifies Experiment 1, “2” denotes Experiment 2. “Control” refers to the normal treatment condition, wherein tobacco seedlings were irrigated with 1/2 Hoagland nutrient solution. “Salt” describes the salt stress treatment condition, which involves supplementing the 1/2 Hoagland nutrient solution with 100 mM NaCl. “Root” indicates that the sampling site for these experiments was the root of the tobacco seedlings.

In a prior study, we performed transcriptomic analysis on the roots of N. tabacum variety Zhongyan 100 under 100 mM NaCl salt treatment (Mao et al., 2021). Leveraging this dataset, the current study explores the gene expression variations across the tobacco Shaker family members. The findings indicate elevated expression levels of Group I members *NKT1A* and *NKT1B* in the roots of tobacco seedlings, in contrast to the lower expression levels of *NKT2A* and *NKT2B*. Similarly, Group II members *NKT3A*, *NKT3B*, *NKT4A*, and *NKT4B* displayed low expression levels. Group III analysis showed low expression levels for *NKT5A* and *NKT5B*, with slightly higher levels for *NKT6A* and *NKT6B*. Notably, Group IV members *NtKC1A* and *NtKC1B*, along with Group V members *NtSKOR1A*, *NtSKOR1B*, *NtSKOR2A*, *NtSKOR2B* and *NtGORKA*, exhibited higher expression levels, while *NtGORKB* was comparatively lower. Post-salt stress treatment comparison revealed that the expression levels of Group V outward rectifying potassium channel members *NtSKOR1A* and *NtSKOR1B* were significantly increased due to salt stress. This suggests a potential involvement of these two genes in the ion transport process under salt treatment conditions. Specifically, *NtSKOR1A* and *NtSKOR1B* were up-regulated by 1.31 and 3.18 times, respectively, compared to pre-treatment levels. Given the higher expression increase of *NtSKOR1B* compared to *NtSKOR1A*, it is posited that *NtSKOR1B* may play a more significant role in this process (**Figure 4**).

### 3.5 Expression pattern analysis of *NtSKOR1B* in *N. tabacum* under salt treatment

**Figure 5:**
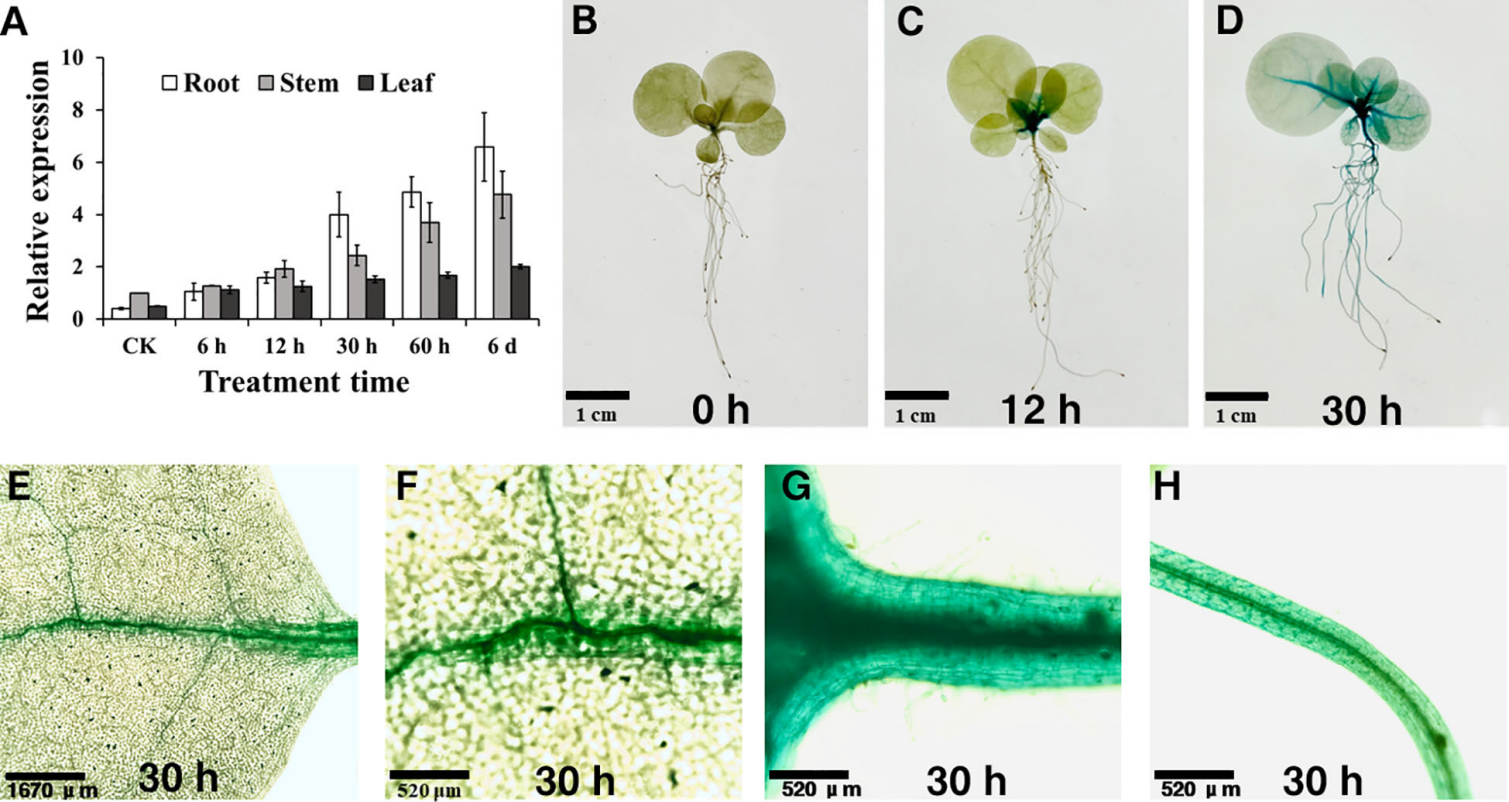
*NtSKOR1B* expression analysis in *N. tabacum* under salt treatment. **(A)** RT-qPCR Analysis of *NtSKOR1B* Gene Expression in Tobacco Seedlings Under Salt Stress. **(B–D)** Time-course GUS Staining in Leaves, Veins, Stems, and Roots at 0h, 12h, and 30h Post-Salt Treatment. **(E–H)** GUS Staining of Leaves, Veins, Stems, and Roots 30h Post-Salt Treatment.

To further investigate *NtSKOR1B* expression changes under salt stress, RT-qPCR was conducted. Results indicated a significant upregulation of *NtSKOR1B* expression in roots with prolonged salt exposure, reaching 9.98-fold after 30 hours compared to the initial level. While *NtSKOR1B* expression in stems and leaves was lower than in roots, it also exhibited a gradual increase over the duration of salt treatment (**Figure 5A**).

A homozygous Zhongyan 100 transgenic line, with the *NtSKOR1B* promoter fused to the GUS reporter gene, was then developed. GUS histochemical staining for the *NtSKOR1B* promoter was consistent with the RT-qPCR findings. GUS staining revealed negligible activity in tobacco seedlings without salt treatment (**Figures 5B–D**). However, GUS activity emerged in the roots, stems, and leaf veins, becoming pronounced after 12 hours and intensifying after 30 hours of salt treatment. This indicates that under standard cultivation conditions, *NtSKOR1B* expression in tobacco seedlings is minimal. Following salt exposure, expression markedly rises in the root cortex, stems, and leaf veins’ vascular tissues, escalating with prolonged treatment (**Figures 5E–H**).

### 3.6 Phenotypic analysis of *NtSKOR1B* knockout mutants under salt treatment

**Figure 6:**
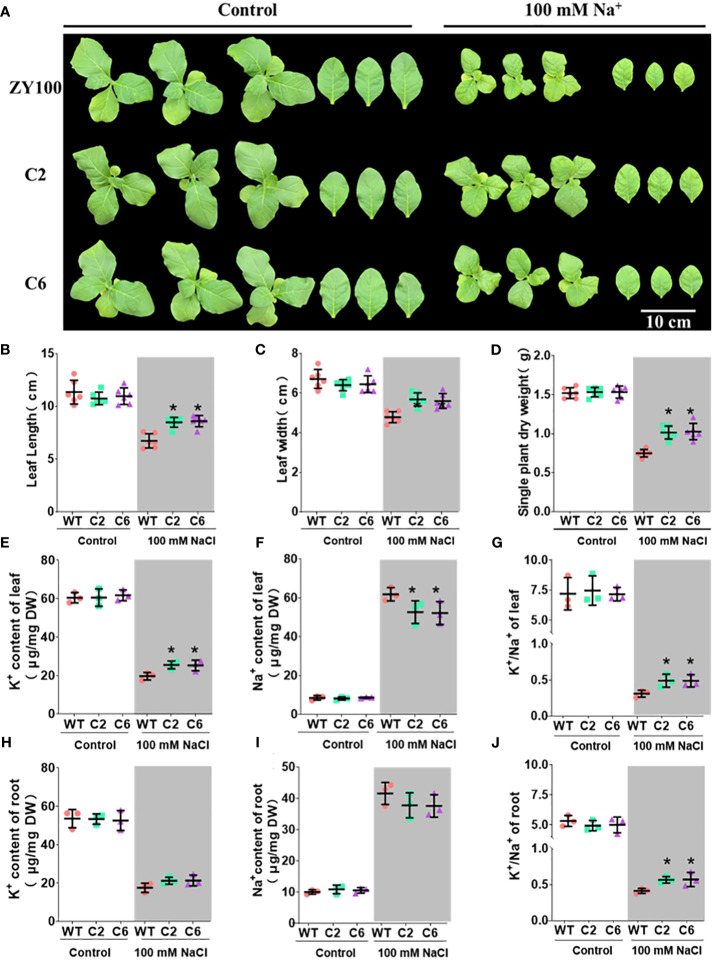
Phenotypic analysis of *NtSKOR1B* knockout mutants under Salt Stress. **(A)** Comparison of Wild-Type and *NtSKOR1B* Knockout mutants Phenotypes Under Control and Salt-Stress Conditions. **(B–J)** Changes in Leaf Length, Leaf Width, Biomass, Leaf and Root K^+^ and Na^+^ Contents, and K^+^/Na^+^ Ratios in Wild-Type and *NtSKOR1B* Knockout mutants Under Control and Salt Stress. “*” represents significant difference compared with wild type, (p<0.05).

To elucidate *NtSKOR1B*’s function under salt stress, two distinct homozygous knockout lines with varied editing methods were exposed to salt treatment (see Materials and Methods for construction and editing details). Under salt stress, *NtSKOR1B* knockout lines demonstrated higher biomass, increased leaf length, and greater leaf width than wild types, with enhancements of 34.06%-39.24%, 15.87%-21.63%, and 14.89%-17.02%, respectively (**Figures 6A–D**). Knockout of *NTSKOR1B* led to increased K^+^ levels in both leaf and root tissues of tobacco seedlings over wild type, showing increases of 22.64%-25.47% and 18.06%-21.02%, with significant differences in aerial tissues (**Figures 6E, H**). Furthermore, *NTSKOR1B* knockout reduced Na^+^ levels in both leaf and root tissues compared to wild types, with reductions of 9.83%-11.48% and 7.52%-7.95%, notably in aerial tissues (**Figures 6F, I**). The *NtSKOR1B* knockout mutants markedly increased the plant’s K^+^/Na^+^ ratio (**Figures 6G, J**). Under control conditions, no significant differences were observed in these physiological indicators or ion levels (**Figures 6A–J**).

## 4 Discussion

Initially, genetic research concentrated on leveraging positive regulatory genes and transgenic technology to elucidate gene-mediated biological process control and its applications in agriculture and related sectors. The advent of CRISPR gene editing technology has redirected research emphasis towards more precise and efficient gene modification techniques, particularly for enhancing crop stress resistance. Recently, negative regulatory genes, or genes exerting inhibitory effects, have emerged as focal points in research aimed at bolstering crop resistance to stress. Investigating these genes is pivotal for pinpointing critical factors that enhance crop survival in harsh conditions, underpinning sustainable agricultural development. Using tobacco as a case study, this research initially pinpointed a negative regulatory gene impacting salt stress resistance. Under salt stress conditions, this gene’s expression can lower the potassium-to-sodium ratio, diminish plant biomass, and weaken salt stress resistance. This suggests that CRISPR-mediated gene editing could markedly enhance crop salt stress tolerance, offering fresh strategies and targets for agricultural enhancement. This discovery not only enriches our comprehension of plant responses to salt stress mechanisms but also underscores the potential of gene editing technology in advancing agricultural science.

### 4.1 The *NtSKOR1B* gene displays diverse expression patterns and functions in different species, suggesting varied roles depending on its expression sites

**Figure 7:**
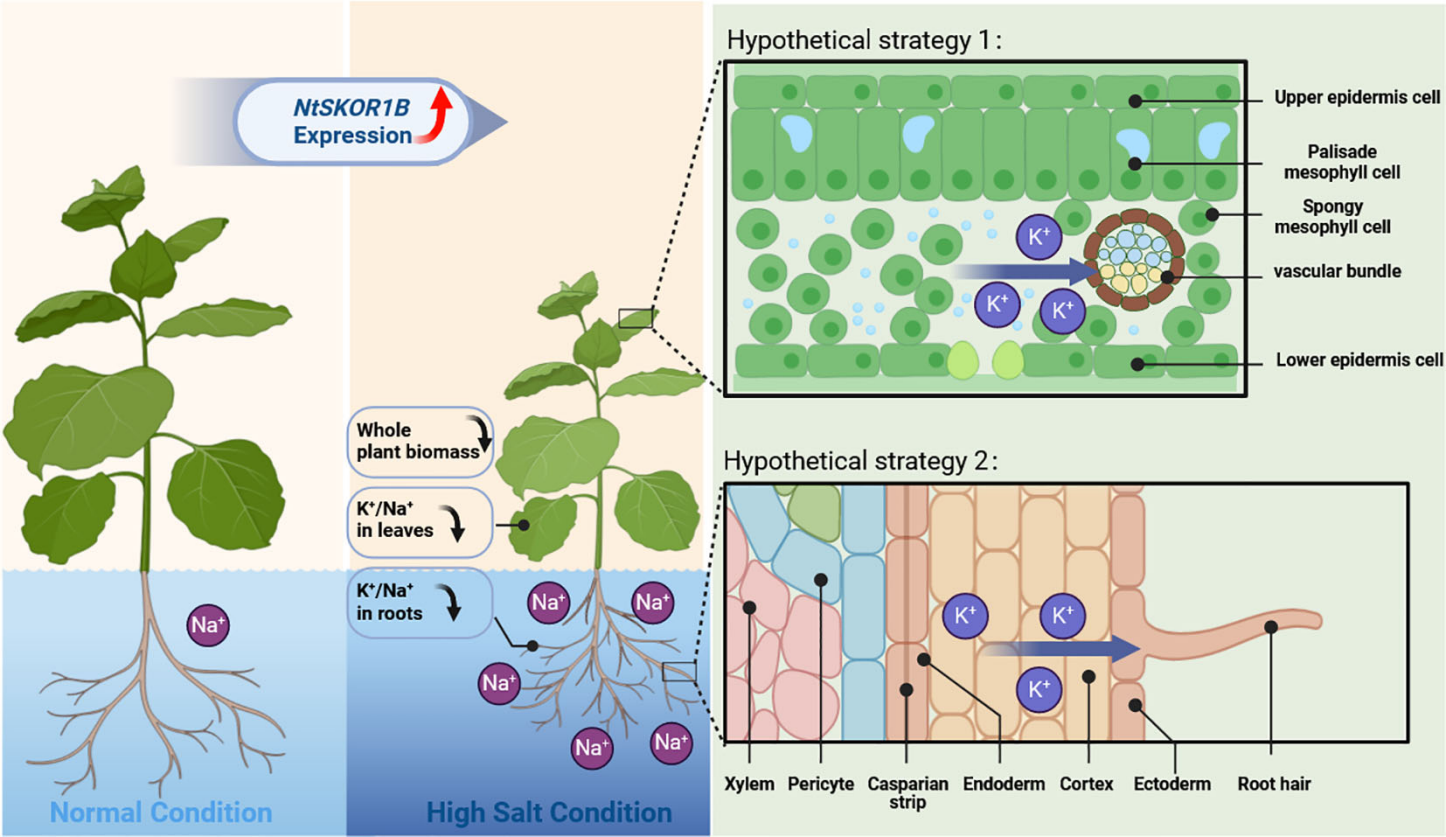
Schematic Representation of *NtSKOR1B* Gene Function in Response to Salt Treatment. The dark blue arrow indicates a decrease in value, while the red arrow shows the direction of K^+^ movement.

Gene expression varies across species and tissues, reflecting gene regulation complexity and biological adaptive evolution (Hill et al., 2021). Current studies demonstrate that the outward rectifying K^+^ channel SKOR is pivotal in regulating intracellular K^+^ balance through mediating cytoplasmic K^+^ efflux, significantly influencing plant responses to salt, drought, and nutritional stresses. The SKOR analogue, OsK5.2, part of the rice Shaker family, resides within the root vascular bundle. During salt stress, OsK5.2 expression boosts K^+^ efflux from the root vascular bundle to the xylem, elevating plant K^+^ levels and thus enhancing salt tolerance through an increased K^+^/Na^+^ ratio. Furthermore, OsK5.2’s presence in guard cells amplifies K^+^ efflux under salt stress, leading to stomatal closure, diminished water loss, and reduced salt buildup, which in turn decreases Na^+^ transport to leaves(Nguyen et al., 2017). In summary, this potassium channel positively influences plant salt tolerance by ensuring a superior K^+^/Na^+^ balance(Zhou et al., 2022). The *NtSKOR1B* outward potassium channel, part of the tobacco Shaker family, is located in the root cortex and leaf vascular bundles. Under salt stress, plants activate K^+^ channels in root cortex cells, leading to K^+^ loss. Outward K^+^ channels play a crucial role in this process (Wakeel, 2013; Demidchik, 2014). *NtSKOR1B* expression speeds up K^+^ efflux, hindering K^+^ level maintenance. K^+^ primarily move from leaves to other plant parts via the phloem. *NtSKOR1B* is found in leaf vascular bundles, not in guard cells. Under salt stress, leaf vascular bundles accelerate K^+^ loss. Analysis revealed that tobacco with *NtSKOR1B* knockout exhibited a higher K^+^/Na^+^ ratio and increased salt tolerance under salt stress (**Figure 6**). Analysis of expression sites (**Figures 5E–H**) shows *NtSKOR1B*’s abundant expression in the root cortex. Knocking out *NtSKOR1B* reduces K^+^ efflux under salt stress. *NtSKOR1B*, also expressed in leaf veins and stem vascular bundles, reduces K^+^ loss in leaves when knocked out. This collectively boosts the plant’s K^+^/Na^+^ ratio and the salt tolerance of tobacco seedlings (**Figure 7**). This study revealed that NtSKOR1B is involved in the negative regulation of salt tolerance in tobacco seedlings, which lays the foundation for further investigation of the function of this gene in intracellular K^+^ and Na^+^ transport in plant wounding fluid and different leaf positions.

### 4.2 The Shaker K^+^ channel member *NtSKOR1B* may be involved in a complex regulatory network

The complexity of gene product interactions and tissue-specific expression patterns, adapted to environmental and physiological demands, are crucial for biological evolution and functional diversity. Shaker K^+^ channels, prevalent in plants, regulate the transmembrane transport of K^+^, playing critical roles in stress response, growth, development, and electrical signal transmission. Arabidopsis *AKT1*, a classic example of a phosphorylation-regulated Shaker K^+^ channel, is activated by CBL1/9-CIPK23 phosphorylation (Wang et al., 2016). Recent studies have elucidated this regulatory pathway further, revealing how plants modulate the plasma membrane CBL1/9-CIPK9/23-*AKT1* in response to external K^+^ levels (Li et al., 2023). Gene expression involves intricate regulatory processes. This study revealed that *NTSKOR1B* negatively influences the salt tolerance of tobacco seedlings. Bioinformatics analysis identified conserved domains within this gene family related to voltage sensing, K^+^ absorption, and the Ankyrin repeat domain for protein-protein interactions. Specifically, the Ankyrin repeat domain in Arabidopsis thaliana can interact with CIPK family proteins to regulate gene expression and respond to environmental stress (Sedgwick and Smerdon, 1999; Wei and Li, 2009). Furthermore, the promoter region is rich in elements tied to stress response, hormonal signals, and growth, such as MYB for drought, cold, and salt stress responses (Yang et al., 2012; Li et al., 2019; Wang et al., 2022), and the hormone response element ABRE (Gomez-Porras et al., 2007). These findings suggest that *NtSKOR1B* is regulated by a complex gene network, offering insights for further detailed exploration of its regulatory mechanisms through techniques such as transcriptome analysis and yeast two-hybrid assays.

### 4.3 *NtSKOR1B* may influence Na^+^ absorption and transport in plants under salt stress by altering K^+^ transport mechanisms

Under salt stress, excessive Na^+^ competes with K^+^ for plasma membrane uptake sites, inhibiting K^+^ absorption and disrupting plant tissue metabolism (Mulet et al., 2023). Excessive Na^+^ influx leads to cell membrane depolarization, reducing the driving force for K^+^ uptake. Consequently, plants in high salinity environments inevitably suffer from chronic K^+^ deficiency. A stable K^+^/Na^+^ ratio is critical for salt tolerance (Horie et al., 2009; Ordonez et al., 2014). Thus, the regulation and distribution of K^+^ are essential for balancing the ionic imbalance caused by Na^+^. K^+^ activates plasma membrane H^+^-ATPase, crucial for cell functioning (Weng et al., 2020). Plasma membrane H^+^-ATPase creates an electrochemical gradient, powering the Na^+^/H^+^ antiporter to exchange H^+^ for Na^+^, reducing Na^+^’s toxic effects (Xie et al., 2022). This study revealed that *NtSKOR1B* knockout in *N. tabacum* L. seedlings under salt stress leads to increased K^+^ and decreased Na^+^ levels, solely at the ionic content level. Further research into Na^+^ transporters, proton pump activities, and expression levels will enhance understanding of *NtSKOR1B*’s role in regulating the K^+^- Na^+^ relationship under salt stress.

### 4.4 Perspectives on the study of negatively regulated genes for salt tolerance

A comprehensive exploration of plant adaptation to salt stress, particularly via analyzing genes that negatively regulate salt tolerance, illuminates the molecular responses of plants to environmental stresses. In response to salt stress, these genes modulate gene expression, preserve ion equilibrium, boost osmoregulator synthesis, fortify antioxidant defenses, and activate distinct signaling pathways. These mechanisms enable plants to regulate ion uptake and distribution, synthesize protective molecules for osmotic balance, and augment antioxidant systems for cellular protection against oxidative stress. Furthermore, the activation of signal transduction pathways triggers a cascade of adaptive and defensive responses in plants. Such research not only enhances our comprehension of plant adaptation mechanisms to environmental stress but also lays the scientific groundwork for developing salt-tolerant crops, pivotal for agricultural productivity and ecological restoration.

## Data availability statement

The original contributions presented in the study are included in the article/**Supplementary Material**, further inquiries can be directed to the corresponding author/s.

## Author contributions

GY: Conceptualization, Data curation, Formal analysis, Investigation, Methodology, Software, Validation, Visualization, Writing – original draft, Writing – review & editing. TN: Conceptualization, Data curation, Formal analysis, Investigation, Methodology, Software, Validation, Visualization, Writing – original draft, Writing – review & editing. OH: Writing – review & editing. CS: Investigation, Writing – review & editing. XS: Investigation, Methodology, Writing – review & editing. FX: Investigation, Methodology, Writing – review & editing. YW: Methodology, Resources, Writing – review & editing. ZZ: Methodology, Resources, Writing – review & editing. YN: Methodology, Resources, Writing – review & editing. HL: Conceptualization, Funding acquisition, Methodology, Project administration, Resources, Supervision, Writing – original draft, Writing – review & editing. QW: Conceptualization, Funding acquisition, Methodology, Project administration, Resources, Supervision, Writing – original draft, Writing – review & editing.
