# Supplementary material for: Genome-wide identification of Shaker K+ channel family in Nicotiana tabacum and functional analysis of NtSKOR1B in response to salt stress
Source: Front Plant Sci. 2024 Apr 10;15:1378738. doi: 10.3389/fpls.2024.1378738 (PMC11039879; doi:10.3389/fpls.2024.1378738)
Supplement: Supplementary file 1 [file DataSheet_1.docx]

Table S1: Primers used for gene expression analysis and plasmid construction.

| Primer Names | Primer sequence | Use |
| --- | --- | --- |
| *NtSKOR1B*pro-1F | TCCCAATCTAACAAAACTGC | Used to amplify *NtSKOR1B* promoter sequence |
| *NtSKOR1B*pro-1R | CTTCAACCGCACTACAAAAT |  |
| *NtSKOR1B*pro-2F | *CCC*AAGCTTTCCCAATCTAACAAAACTGC | *Hind III* and *Sma I* restriction sites and corresponding protective bases were introduced at both ends of the *NtSKOR1B* promoter to construct the pBI101-*NtSKOR1B*pro vector. |
| *NtSKOR1B*pro-2R | *TCC*CCCGGGCTTCAACCGCACTACAAAAT |  |
| *NtSKOR1B*pro-3F | CGAGAGATGAGGAATGGTAAC | For positive identification of promoter material |
| *NtSKOR1B*-1F | ATGTCGATGATGAGGAGAGAAG | Used to amplify the *NtSKOR1B* coding region sequence |
| *NtSKOR1B*-1R | TCAAGTTGTTTCACTGATCAAGTAC |  |
| *NtSKOR1B*-Crispr-58F | GATTGAGGGAAATGATATTCGTAA | *NtSKOR1B* editing primers |
| *NtSKOR1B*-Crispr-58R | AAACTTACGAATATCATTTCCCTC |  |
| *NtSKOR1B*-Crispr-9734F | CTTATCTACATCCCCTTCCCT | *NtSKOR1B*-Crispr material-specific primer detection |
| *NtSKOR1B*-Crispr-10293R | CATATTGTAATCGCAAGTGATC |  |

Note: The underlined sequence is the restriction site, and the italicized sequence is the protective base.


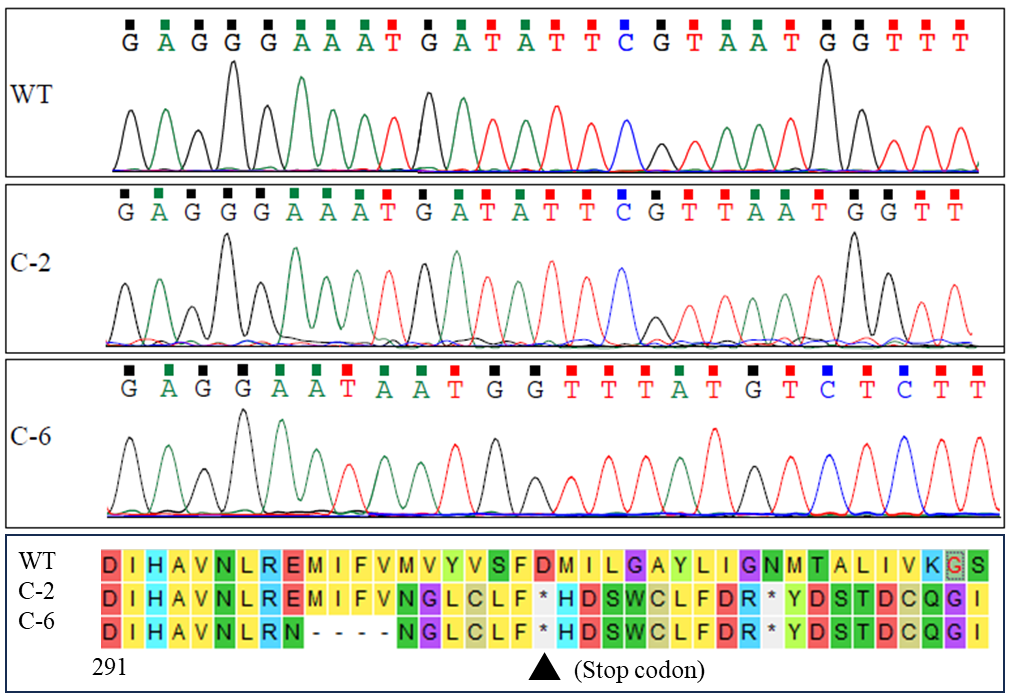


Figure S1: Gene editing locations and protein translation in two *NtSKOR1B* gene knockout mutants.
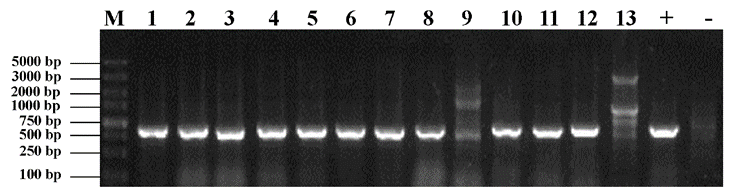


Figure S2: PCR identification of *N. tabacum* L. *ProNtSKOR1B::GUS* transgenic plants.

Note: M, Marker DL5000; ^+^, pBI101-*NtSKOR1BB*pro plasmid; -, Zhongyan100.
